# Broadening the Phenotypic Spectrum of Forkhead Box N1 Gene Mutations

**DOI:** 10.7759/cureus.94410

**Published:** 2025-10-12

**Authors:** James Brader, Rachael O'Brien, Clare Rees

**Affiliations:** 1 Internal Medicine, Frimley Park Hospital, Frimley, GBR; 2 Immunology, Frimley Park Hospital, Frimley, GBR; 3 Haematology, Frimley Park Hospital, Frimley, GBR

**Keywords:** cd4+ lymphopenia, foxn1, genetic screening, hypogammaglobulinaemia, primary immunodeficiency disease

## Abstract

A man in his 50s develops signs of immune dysfunction (recurrent chest infections and new-onset chronic diarrhoea), alongside a history of multiple distinct malignancies and nail dystrophy. Immunological testing reveals T-cell lymphopenia and hypogammaglobulinaemia. Whole genome sequencing reveals a heterozygous c.1465del mutation in the *FOXN1 *(forkhead box N1) gene. This case suggests that previously healthy carriers of heterozygous *FOXN1* mutations may be at risk of developing immune dysfunction in later life. Potential reasons for the severe and delayed phenotype include a dominant-negative effect of the resultant protein (p.Gln489ArgfsTer61), age-related involution of the thymus and previous chemoradiotherapy. Further studies on heterozygous *FOXN1* mutations are required to clarify their clinical significance and inform evidence-based management approaches.

## Introduction

*FOXN1* (forkhead box N1) is the master transcription factor essential for thymic epithelial cell (TEC) development [1], thus playing a crucial role in T-cell maturation and selection, allowing the immune system to respond to foreign antigens whilst remaining tolerant to self [2]. Outside of the thymus, it is involved in growth and differentiation of epithelial cells within the epidermis, hair follicles and nail beds [2]. 

*FOXN1* mutations can be subcategorised by zygosity. Homozygous mutations typically result in biallelic loss of function and the “nude/severe combined immunodeficiency (SCID)" phenotype characterised by complete athymia, alopecia and nail dystrophy [3]. Compound heterozygous mutations have been associated with a range of features, including SCID features without typical nail or hair changes [4]. Heterozygous mutations have resulted in variable phenotypes, with reports of the SCID or CID (combined immunodeficiency) phenotype in an early age [3]. There are also reports of nail dystrophy and infrequent reports of hair loss or thinning [3]. The variability in phenotypes may reflect environmental factors or modifier genes. 

Heterozygous variants could be under-recognised clinically because features such as alopecia and nail dystrophy may be absent [3]. Furthermore, heterozygous mutations are associated with milder degrees of lymphopenia when compared to homozygous mutations [2]. This lymphopenia tends to be more pronounced in infancy and improves over time [2], potentially leading to missed diagnoses in patients without adequate newborn screening. This case report is regarding a heterozygous mutation that presents later in adulthood.

## Case presentation

The patient is a man in his late 50s being investigated by the immunology team due to concerns about a primary immunodeficiency. His past medical history is shown in Table 1. 

**Table 1:** The patient’s past medical history. R-CODOX-M/IVAC: rituximab, cyclophosphamide, Oncovin (vincristine), doxorubicin, methotrexate, ifosfamide, etoposide, and cytarabine; CMR: complete metabolic response; CT: computed tomography; EBV: Epstein-Barr virus; NHL: non-Hodgkin lymphoma; SCC: squamous cell carcinoma; DLBCL: diffuse large B-cell lymphoma; Pola-R-CHP: polatuzumab vedotin with rituximab, cyclophosphamide, and prednisolone.

Approximate age of onset	Description of medical history
Longstanding	Nail dystrophy
50	Stage IVB Burkitt lymphoma with immunoglobulin heavy chain gene/MYC translocation t(8;14) that was treated with R-CODOX-M/IVAC chemotherapy to CMR.
51	Lung nodule first noted in the right upper lobe. Biopsy results at the time showed acute inflammation only. The nodule was presumed to be infective in cause. The nodule has been under surveillance since with CT thorax scans showing no new concerning features.
53	Anal condylomata acuminata, since resolved.
53	EBV-derived mucocutaneous ulcer of the nose treated with rituximab.
54	Development of recurrent chest infections that have meant the requirement for prophylactic medications including doxycycline and more recently co-trimoxazole. No sputum culture results have been obtained.
55	Low-grade B-cell NHL with features most in keeping with marginal zone subtype diagnosed on cervical lymph node biopsy four years prior, for which he has received active monitoring.
55	SCC of the lip that was surgically excised.
56	Moderately differentiated SCC of the anal canal treated with concurrent chemoradiotherapy (capecitabine and mitomycin C, 50.4 Gy in 28 fractions) to CMR.
57	Stage IE DLBCL of mandible (non-germinal centre subtype, no MYC rearrangement on fluorescence in situ hybridisation) that was treated with six cycles of Pola-R-CHP and consolidative radiotherapy to CMR.
57	New-onset chronic diarrhoea.

Immunological tests were sent to investigate a possible primary immunodeficiency; the results are shown in Table 2 and Table 3. T_1_ was immediately after the patient’s first cycle of R-CODOX-M/IVAC chemotherapy for stage IVB Burkitt Lymphoma (see Table 1). T_2_ was approximately seven weeks following chemoradiotherapy for SCC of the anal canal (see Table 1). T_3_ was in between cycles of Pola-R-CHP for DLCBL (see Table 1). Table 2 shows T-cell deficiency, appearing to affect CD4 cells more than CD8 cells; it also shows CD19 depletion at T_3_, which is likely due to rituximab. Table 3 shows hypogammaglobulinaemia, with significant IgM depletion at all time points tested, assumed to be due to rituximab. The normal levels of IgG and IgA at T_1_ suggest that the antibody deficiency was not present prior to starting any chemotherapy regimens.

**Table 2:** Lymphocyte subset testing, showing T-cell deficiency.

Lymphocyte subset	Absolute count at time T_2 _(10^9^/L)	Absolute count at time T_3_ (10^9^/L)	Laboratory normal reference range (10^9^/L)
CD3	0.48	0.42	0.7-2.1
CD8	0.22	0.22	0.2-0.9
CD4	0.23	0.18	0.3-1.4
CD16/56	0.11	0.12	0.09-0.6
CD19	0.14	<0.025	0.1-0.5

**Table 3:** Immunoglobulin (Ig) subset testing, showing hypogammaglobulinaemia. IgG: Immunoglobulin G; IgA: Immunoglobulin A; IgM: Immunoglobulin M.

Immunoglobulin	Levels at T_1 _(g/L)	Levels at T_2 _(g/L)	Levels at T_3_ (g/L)	Laboratory normal reference range (g/L)
IgG	7.2	3.2	3.6	6-16
IgA	2.05	0.8	0.66	0.8-4.0
IgM	<0.17	0.11	<0.05	0.5-2.0

**Figure 1:**
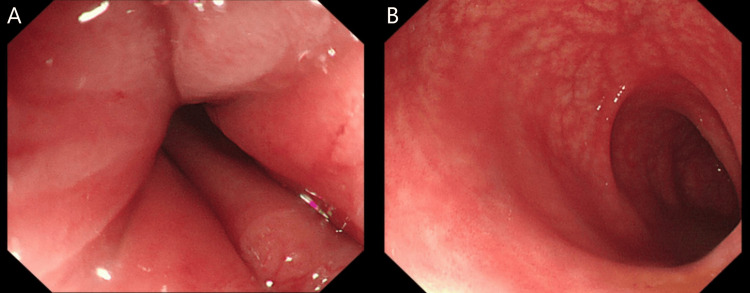
Images from the patient's flexible sigmoidoscopy. A: Normal appearances of the rectum. B: Normal appearances of the sigmoid colon.

**Figure 2:**
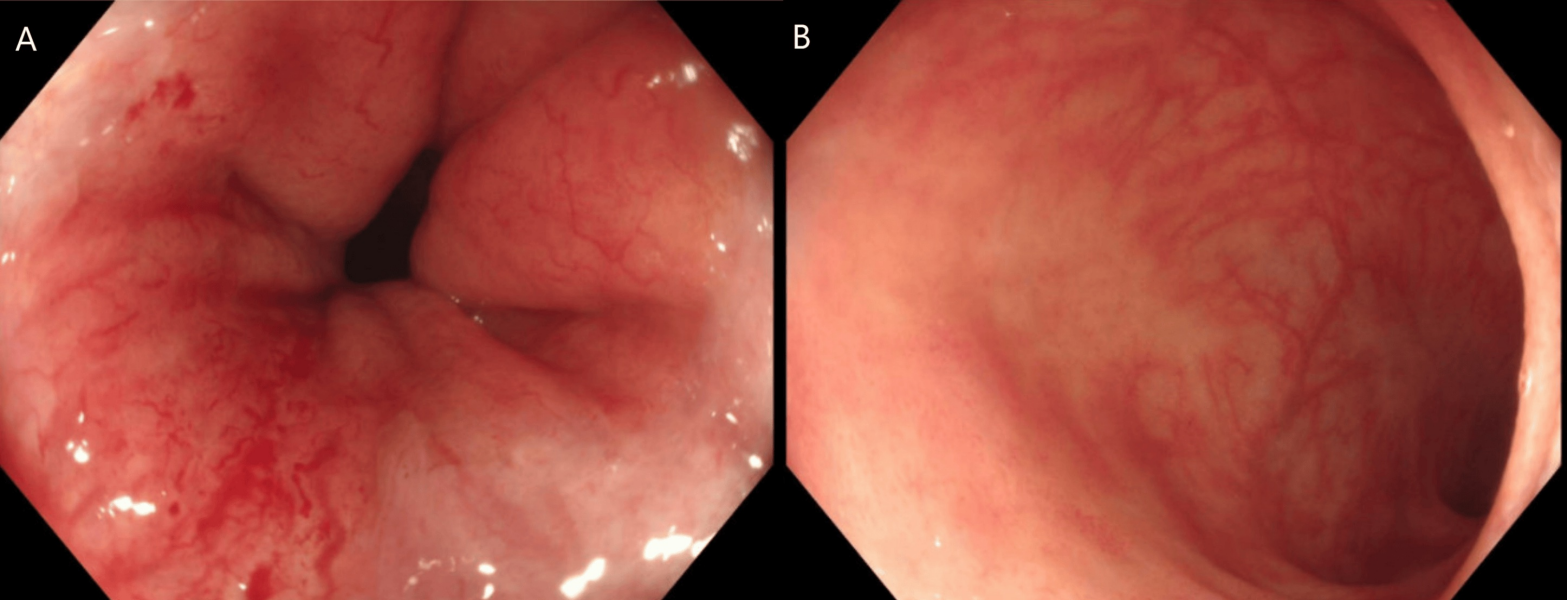
Images from the patient's colonoscopy. A: Oedematous, erythematous and petechial mucosa within the rectum, consistent with radiation proctitis. B: Normal appearances of the terminal ileum.

Table 4 shows the results of tests investigating the patient’s new-onset chronic diarrhoea, showing that an infective cause for the diarrhoea was not found. Figure 1 shows images from the patient's flexible sigmoidoscopy (performed approximately three months following the onset of chronic diarrhoea), whilst Figure 2 shows images from the patient's colonoscopy (performed one year following the flexible sigmoidoscopy). In both cases, macroscopic and microscopic appearances were deemed not suggestive of inflammatory bowel disease. The colonoscopy showed evidence of radiation proctitis (likely as a result of previous radiotherapy to the anal canal).

**Table 4:** The results of investigations performed to investigate the patient’s new-onset chronic diarrhoea. PCR: polymerase chain reaction; sp.: species; Ab: antibody; Ag: antigen.

Category	Test	Result
Faecal culture and molecular diagnostic testing PCR	Cryptosporidium sp.	Negative
	Giardia sp	Negative
	Salmonella sp.	Negative
	Shigella sp.	Negative
	Campylobacter sp.	Negative
	Verotoxin-producing Escherichia coli O157	Negative
	Clostridiodes difficile	Negative
Viral and parasitic serological testing	Cytomegalovirus IgG	Negative
	Cytomegalovirus deoxyribonucleic acid	Negative
	Adenovirus (via PCR)	Negative
	Toxoplasma gondii IgG Ab	Negative
	Hepatitis B surface antigen	Negative
	Hepatitis B core Ab	Negative
	Hepatitis C Ab	Negative
	Human immunodeficiency virus types 1 and 2 Ab and p24 Ag	Negative
	EBV viral load	Detected 16 months prior to this report but was not detected six months ago
Endoscopy	Flexible sigmoidoscopy (see Figure 1)	Normal appearances of the sigmoid colon, rectosigmoid junction and rectum. Biopsy appearances showed very mild inflammation and non-specific changes, possibly suggestive of post-infectious aetiology.
	Colonoscopy (see Figure 2)	Radiation proctitis in the distal rectum. Biopsies taken from right and left colon showed mild active chronic inflammation. Macroscopic and microscopic appearances were deemed not consistent with inflammatory bowel disease.

Due to the clinical history of multiple malignancies and infections, alongside immunological testing showing hypogammaglobulinaemia and T-cell lymphopenia, it was considered that he might have an inborn error of immunity. Whole genome sequencing was therefore performed. The patient was 58 when this was performed. Results showed a heterozygous *FOXN1* mutation, specifically c.1465del p(Gln489ArgfsTer61). No other potentially relevant genetic variants were found.

The patient is now under regular monitoring for malignancies. Potential infections are to be fought with the continuation of prophylactic antibiotics. The patient is due to commence on immunoglobulin replacement therapy, with the hope of reducing infection frequency. 

The patient has a first-degree relative with similar nail changes. There are no reports of family members suffering from recurrent infections. He and his family have been referred to the clinical genetics team for further consideration of the implications of the *FOXN1* mutation on his wider family.

## Discussion

Table 5 shows a summary of previously published reports of the c.1465del p(Gln489ArgfsTer61) *FOXN1* mutation.

**Table 5:** Previous reports of the c.1465del p.(Gln489ArgfsTer61) FOXN1 mutation summarised in table form. TRECs: T-cell receptor excision circles; T-/loB+NK+: T-cell negative/low, B-cell positive, NK-cell positive; HSCT: haematopoietic stem cell transplantation.

Author	Zygosity	Summary
Du et al., 2019 [4]	Heterozygous	Two patients with the same heterozygous mutation were described as "healthy, no recurrent infections"; however, the ages of the patients were not specified, nor was the follow-up period. One of the two patients was reported to have alopecia. Immunological parameters were not reported.
Du et al., 2019 [4]	Compound heterozygous (c.1288C>T/c.1465delC)	Mutation was picked up following abnormal newborn screening for SCID, in which TRECs were absent/very low. Diagnosed with T-/loB+NK+ SCID. The patient received HSCT and gammaglobulin therapy. Following these interventions, they were reported as "healthy, with no recurrent infections". They had normal hair and nails.
Fuentes et al., 2022 [5]	Heterozygous	Mutation was picked up following abnormal newborn screening for SCID in which TRECs were absent/very low. Initial immune investigations showed severe T-cell lymphopenia, particularly affecting CD4+ and CD8+ cells, leading to a diagnosis of T-B+NK+ SCID. The patient remained clinically well with watchful waiting and monitoring of immunological tests. Over the course of the first 18 months of life, CD4+ and CD8+ counts generally improved but remained low relative to normal ranges.

Heterozygous *FOXN1* mutations show variable immunological presentation. Some mutations present with T-cell lymphopenia detectable at newborn screening, while others may not be detected until later [6]. A typical pattern described is of CD4+ T-cell counts normalising through age through homeostatic proliferation, while CD8+ T-cell lymphopenia often persists into adulthood [6]. Lymphocyte subsets in this case did show T-cell lymphopenia, however, curiously affecting CD4+ counts more than CD8+ counts. This atypical pattern may reflect individual variation or age-related changes not fully characterised in the literature, but results must be contextualised in relation to recent chemotherapy. At time T_2_, recent administration of chemoradiotherapy (including capecitabine and mitomycin C) seven weeks prior would likely have been key in depleting T-cell counts. At time T_3_ (following Pola-R-CHP cycles), a similar event could have occurred, with more severe B-cell depletion associated with this chemotherapy regimen, in particular due to the rituximab component. It is likely that within the context of thymic dysfunction, immunological recovery following chemotherapy would be significantly reduced. The clustering of malignancies and infections in this case over a short time period supports the hypothesis of immune dysfunction, whilst also emphasising the importance of longitudinal monitoring for patients with *FOXN1* mutations, particularly following cytotoxic therapy. 

The T-cell lymphopenia and hypogammaglobulinaemia observed in this patient would be expected to compromise tumour surveillance alongside infection control mechanisms, whilst also explaining the susceptibility to EBV-driven pathologies including Burkitt lymphoma and mucocutaneous ulceration.

Mechanistic studies have shed some light on the reasons for the varied clinical phenotypes observed with different heterozygous *FOXN1* mutations; phenotype severity may depend on whether the mutant allele produces a protein with dominant-negative properties [7], actively interfering with the function of the wild-type FOXN1 protein. FOXN1’s transcriptional activity is related to its formation of nuclear condensates, where gene regulation is orchestrated [8]. Within these condensates, the C-terminal region of FOXN1 modulates diffusion velocity and the binding to proximal gene regulatory regions [8]. Studies of the heterozygous c.1370delA mutation have shown that it leads to C-terminal truncation [8]. The resultant mutant FOXN1 protein lacks transcriptional activity, whilst also displacing wild-type FOXN1 from condensates, leading to a dominant negative effect, with resultant athymia and severe lymphopenia [8].

For the mutation described in this case, c.1465del represents a deletion of a single nucleotide at position 1465 within the coding DNA sequence. This results in a frameshift, leading to an altered protein denoted as p.(Gln489ArgfsTer61), in which the normal glutamine at position 489 is replaced by an arginine due to a frameshift, and a premature stop codon is introduced 61 amino acids downstream. Similar to the c.1370delA mutation, this frameshift mutation and resultant premature stop codon lead to truncation of the FOXN1 protein. Mechanistically, this truncation affects the C-terminal transactivation domain while preserving the DNA binding domain, eliminating critical acidic residues required for transcriptional activation and resulting in a dominant-negative effect that suppresses wild-type FOXN1 function [7]. The resultant truncated protein retains only 5% transcriptional activity [7], with dominant-negative effects observed in human in vitro models but not reproduced in murine models [7], suggesting species-specific differences. Nonetheless, the dominant-negative effect in humans helps explain the severe phenotype found in this case.

The delayed onset of clinical features in late adulthood, rather than early childhood, may reflect a convergence of factors. Firstly, age-related involution of the thymus is associated with a reduction in TEC numbers alongside *FOXN1* expression [9], leading to increased vulnerability to FOXN1 dysfunction. Secondly, the ongoing displacement of wild-type FOXN1 from nuclear condensates may gradually erode residual TEC function. Thirdly, immune dysfunction was likely accelerated by multiple chemotherapy and radiotherapy courses received, starting nine years ago with R-CODOX-M/IVAC, a myelosuppressive and lymphotoxic regimen leading to T-cell lymphopenia. These factors likely acted together to result in immune dysfunction, with severely compromised capacity for immune recovery, particularly of thymic-dependent T-cell populations.

Whilst HSCT has been used as a management option for patients with *FOXN1* mutations, post-transplant outcomes have indicated that thymus transplants are a more curative option [6]. Unlike HSCT, thymus transplantation addresses the underlying primary defect of thymus epithelial cell dysfunction and has shown promise in previous cases of *FOXN1* mutations [1]. However, this is only from a relatively small number of cases. Importantly, thymus transplantation appears to be highly time sensitive, as delayed diagnosis and intervention may result in severe infections or immune dysregulation prior to immune reconstitution [10]. In this context, programmes such as the United Kingdom National Health Service pilot of whole genome sequencing for all newborns could lead to earlier detection and intervention. Such approaches may, however, identify genetic variants of uncertain significance, such as heterozygous *FOXN1* mutations, where prognosis and the need for monitoring or management remain unclear.

## Conclusions

The mutation identified during this case was associated with significant pathology developed later in life, highlighting the potential value of long-term follow-up and family screening for individuals with similar genetic findings. Further studies should better characterise the long-term immunological consequences of *FOXN1* mutations, which are likely to be strongly linked to resultant protein structure and possible dominant negative mechanisms. As suggested by this case, factors such as age-related involution of the thymus and chemoradiotherapy may exacerbate underlying immune dysfunction, and eventually lead to significant pathology including multiple distinct malignancies and recurrent infections. Further understanding of the implications of *FOXN1* mutations would help decide management options for future patients, with options such as thymus transplantation likely remaining time-sensitive and for severe cases.
